# Combination of Spray-Chilling and Spray-Drying Techniques to Protect Carotenoid-Rich Extracts from Pumpkin (*Cucurbita moschata*) Byproducts, Aiming at the Production of a Powdered Natural Food Dye

**DOI:** 10.3390/molecules27217530

**Published:** 2022-11-03

**Authors:** Priscilla Magalhães de Lima, Gustavo César Dacanal, Lorena Silva Pinho, Samuel Henrique Gomes de Sá, Marcelo Thomazini, Carmen Sílvia Favaro-Trindade

**Affiliations:** Departamento de Engenharia de Alimentos (ZEA), Faculdade de Zootecnia e Engenharia de Alimentos (FZEA), Universidade de São Paulo (USP), Pirassununga 13635-900, SP, Brazil

**Keywords:** encapsulation, colours, pigment, β-carotene, food additive, stability

## Abstract

Reducing waste, using byproducts, and natural food additives are important sustainability trends. In this context, the aim of this study was to produce and evaluate a natural food dye, extracted from pumpkin byproducts, powdered and protected by spray-chilling (SC) and a combination of spray-drying and spray-chilling techniques (SDC). The extract was obtained using ethanol as solvent; vegetable fat and gum Arabic were used as carriers. Formulations were prepared with the following core:carrier ratios: SC 20 (20:80), SC 30 (30:70), SC 40 (40:60), SDC 5 (5:95), SDC 10 (10:90), and SDC 15 (15:85). The physicochemical properties of the formed microparticles were characterised, and their storage stability was evaluated over 90 days. The microparticles exhibited colour variation and size increase over time. SDC particles exhibited the highest encapsulation efficiency (95.2–100.8%) and retention of carotenoids in the storage period (60.8–89.7%). Considering the carotenoid content and its stability, the optimal formulation for each process was selected for further analysis. All of the processes and formulations produced spherical particles that were heterogeneous in size. SDC particles exhibited the highest oxidative stability index and the highest carotenoid release in the intestinal phase (32.6%). The use of combined microencapsulation technologies should be considered promising to protect carotenoid compounds.

## 1. Introduction

Pumpkins are widely cultivated and consumed worldwide. These vegetables are rich in provitamin A carotenoids [1]. However, when these products are processed, only their pulps are used, generating between 18% and 21% wastage [2]. Pumpkin peel represents about 10–40% of these vegetables and is a rich source of provitamin A carotenoids, which also have antioxidant properties [3,4]. Consumption of carotenoids has been associated with various health benefits, including a reduced risk of cataracts and age-related macular degeneration, coronary heart disease, and some cancers [5]. However, due to their high physicochemical instability, the carotenoids are easily degraded in the presence of light, oxygen, metals, and constituents present in the food matrix, as well as when exposed to heat during processing and storage, so it is necessary to use techniques that ensure their protection and, therefore, the integrity and maintenance of their functional properties [6].

Spray-drying (SD) is a traditional encapsulation technique that is widely used in the food industry. This method makes it possible to obtain powders with the desired physical properties, apply variable drying parameters—such as temperature and feed flow—and change the type of carrier material or the pretreatment of the raw material [7,8,9]. One disadvantage of this method is the production of matrix-type structures, so some carotenoids can remain on the surface of the microparticles, making them more likely to undergo oxidation [10]. Therefore, it is assumed that the coating of particles produced by this technique could offer extra protection to carotenoids, in addition to altering their release pattern. Thus, in this work, we propose covering the spray-dried particles with a lipid carrier using the spray-chilling (SC) technique.

The SC technique has been less explored for the encapsulation of food ingredients—both scientifically and commercially; it consists of the atomisation of a mixture, solution, or suspension of an active material and a molten fat carrier within an environment maintained at a temperature below the carrier’s melting point. The melted vegetable fat in contact with cold air produces solid particles [11,12]. Although simple, fast, and cheap, this technique has some disadvantages, such as low encapsulation efficiency and expulsion of the active ingredient during storage when there is recrystallisation of the lipid carrier, in addition to the hydrophobic character of the particles, which renders some applications difficult [12]. On the other hand, it is known that carotenoids are absorbed in the presence of lipids; therefore, their immobilisation in lipid particles should also favour their intestinal absorption, in addition to promoting protection.

Furthermore, the association of the two techniques—something that has been little explored—offers the possibility of better preservation of bioactive components considering the presence of a double-wall coverage. In the literature, there are few studies concerning the production of microparticles combining SD and SC. Fadini et al. [13] reported high protection of functional oils when using combined technologies. Arslan-Tontul and Erbas [14] observed higher thermal and gastric resistance of particles containing prebiotics produced by the combination of SD and SC than of those produced by the individual processes. Pinho et al. [15] observed high protection of carotenoids when using these combined technologies.

Brazil has the largest number of authorised synthetic dyes. However, there has been a change in the global trend, reducing the consumption of foods with synthetic dyes, due to the search for healthier foods [16]. Moreover, some of these synthetic dyes are allergenic, mutagenic, or carcinogenic, and may generate behavioural changes, such as hyperactivity, increasing the concern of health authorities worldwide [17]. Although natural dyes with the same economic and technological advantages as synthetic dyes are not available, there is a high preference for pigments obtained from natural sources, given their nutritional value and health benefits [18].

Thus, the aim of this study was to produce and evaluate a natural food dye composed of microparticles loaded with carotenoid-rich extracts from pumpkin peel and produced by SC and by the combination of SD and SC technologies, with the objective of verifying which encapsulation method would promote greater stability of the bioactive compounds. The microparticles were monitored during storage in terms of size, colour parameters, and carotenoid stability. The physicochemical properties of the microparticles—including morphology, oxidative stability of carotenoids, and release of carotenoids in the digestive system in vitro—were measured. The influence of different carrier materials on the properties of the microparticles was also investigated.

## 2. Results and Discussion

### 2.1. Encapsulation Efficiency, Carotenoid Stability during 90 Days of Storage, and Instrumental Colour Parameters

The main objective of encapsulation is to produce microparticles with good application properties. Factors related to the inlet air temperature, feed flow rates, formulation, and type of carrier material are essential because they affect the microparticles’ characteristics and encapsulation efficiency (*EE*).

The *EE* of microparticles produced through SC and the combined processes is shown in Table 1.

According to the results, the *EE* values after both spraying processes were high, showing that—at least initially—these were not harmful to the carotenoids, which demonstrates greater entrapment of the carotenoids in the microparticles for all treatments used. The SC-20 treatment presented the lowest value, which may have occurred due to a low *EE*, presenting carotenoids on the surface, and the occurrence of degradation. Rodriguez-Amaya [19] showed that the major cause of carotenoid degradation during the processing and storage of food is enzymatic or non-enzymatic oxidation. Isomerisation of trans-carotenoids to cis-isomers discolours food and is promoted by contact with heat treatment, acids, and exposure to light [19]. In the case of SDC particles, the *EE* values were higher, evidencing the protection offered to carotenoids by the double layer of gum Arabic and vegetable fat. Cutrim, Alvim, and Cortez [20], when encapsulating green tea polyphenols by SC, found values similar to those of the present study (83.5%). Gamboa, Gonçalves, and Grosso [21] found values similar to those of our study when encapsulating α-tocopherol by SC using different proportions of inter-esterified fat and hydrogenated soy oil (90–95.8%). Sartori et al. [22] obtained a high *EE* (89–98%) when encapsulating ascorbic acid by SC and using lauric and oleic acid as carrier materials.

Degradation of carotenoids loaded into spray-chilled and double-layer microparticles over the storage period under different conditions presented zero-order kinetics; the results are shown in Table 1. All formulations in all storage conditions presented significant zero-order constants of degradation (*k* = 0.017 to 1.096 μg.g^−1^.s^−1^), indicating an intense and accelerated degradation of carotenoids, as also evidenced by the half-life (*t*_1/2_). The results indicate that carotenoid degradation was highly influenced by the encapsulation method. Therefore, according to these aspects, SDC microparticles showed superior protective capacity, ensuring greater stability of the carotenoids over 90 days, as demonstrated by the half-life data.

When varying the carotenoid ratio in the microparticles, the stability was reduced in formulations with elevated ratios of free extract (SC particles) and spray-dried particles (SDC particles). This may indicate that carotenoids are less protected from environmental conditions in such formulations, since more carotenoid molecules may be present on the surface of the particles and become susceptible to degradation reactions. SC-30 showed the lowest half-life value; this was probably due to the large presence of carotenoids on the surface of the particles, with greater degradation, which can be explained by the retention value, which was the lowest of all treatments.

The carotenoid retention values (CR, %) in the SC particles varied from 51.1% to 65.9%. A possible entrapment of oxygen within the carrier matrix in the process may contribute to the oxidation of carotenoids during storage. In addition, isomerisation can also cause the degradation of these compounds. Furthermore, according to Lima et al. [23], the ethanolic extract of pumpkin peel has a high Fe content, which may have contributed to the greater degradation of carotenoids in the SC samples, since Fe is a pro-oxidant.

However, microparticles obtained via SDC displayed more effective preservation of the carotenoids, indicating that the composition of the carriers impacts the stability of the active components. This result is consistent with the colour change observed for the different samples. According to Provesi, Dias, and Amante [24], light and temperature are the main causes of carotenoid isomerisation. However, Rodriguez-Amaya [25] reported that the main causes of carotenoid degradation during storage are enzymatic and non-enzymatic oxidations caused by the presence of oxygen inside the particles and the carotenoids’ molecular structure. Similar results were found by Pelissari et al. [26]—when encapsulating lycopene with SC, they observed degradation rates of around 60%. Santos et al. [27], when producing nanoparticles loaded with lycopene, reported 50% retention after 14 days of storage at room temperature, indicating the influence of temperature on carotenoids’ stability. Fadini et al. [13] found retention values lower than those found in this study when combining SD and SC techniques to encapsulate fish oil and sacha inchi oil (45–60%). The retention values (CR, %) over the storage period found in this study were higher for the coated particles, indicating that this technique is a good alternative to protect carotenoids.

Colour is another parameter that may be related to the stability of carotenoids, since the higher the total colour difference value, the greater the pigment degradation. The instrumental colour parameters were monitored during 90 days of storage, since evaluations of colorimetric parameters may be considered simple but powerful alternatives for indirect measurement of the pigment contents in food products [28].

The colour brightness coordinate L* indicates the lightness value [29]. SDC-5 microparticles showed higher lightness (L*) compared to the other treatments. This was due to the higher content of hydrogenated vegetable fat, as well as the lower ratio between the concentrations of SD microparticles in their formulations. The value of L* (Table 2) decreased over the storage period; this was related to the way in which the particles absorb light, as confirmed by the increase in average diameter (Table 3).

McClements [30] reported that the increase in particle size caused by aggregation can influence the lightness of samples, affecting their L* value. 

Values of chroma (Table 2)—considered to be a quantitative attribute of colourfulness—indicate the intensity of the colour. The samples with lower concentrations of carrier material had higher chroma values. These values were stable for most samples, with the exception of SC-40 and SDC-15, indicating a decrease in colour intensity, which was expected, since these samples had lower concentrations of carrier material.

Hue angle is considered to be a qualitative attribute of colour, and it is related to the differences in absorbance at different wavelengths. A hue angle of 0° or 360° denotes a red hue, while angles of 90°, 180°, and 270° represent yellow, green, and blue hues, respectively [31]. In the present study, the hue angle lay in the region of red and yellow, between 0° and 90°, indicating that the colour of the product was a yellow hue. After 90 days, there was no significant change in the samples, and the tonality was maintained for all treatments—with the exception of SDC-5 which, at the end of the stability period, showed a tonality tending towards green; this occurred because there was a decrease in the values of a * after 90 days (data not shown).

Another important parameter estimated was the total colour difference (ΔE); according to the results shown in Table 2, the SC-30 and SC-40 particles showed higher values of ΔE when compared to SC-20. This result can be confirmed by the retention of carotenoids in this period and can be attributed to the highest rate of carotenoid oxidation, since they had the lowest concentration of the carrier, so the carotenoids were presumably less protected and more exposed to pro-oxidant factors, such as oxygen and temperature.

The SDC-5 treatment showed greater colour variation. These results are not consistent with the chroma values and carotenoid retention values, which may have been caused by the large variation in L*, which may itself have been caused by the increase in average diameter (i.e., agglomeration of particles), causing a reduction in this parameter and, consequently, a higher ΔE. Haas et al. [32] found similar results to those in our study when studying the influence of particle size on encapsulated carrot carotenoids, observing reduced brightness and increased colour saturation.

However, the other treatments showed lower values, indicating that the coating was effective in protecting the carotenoids, minimising their rapid degradation. However, these values were lower than those found by Pelissari et al. [26] when they encapsulated lycopene via SC and evaluated its stability for 90 days at room temperature with 33% relativity humidity, with ΔE ranging from 25.47 to 67.56. Even though the samples showed differences in ΔE, these values were relatively low, indicating that the encapsulation processes were effective in protecting the colour throughout the storage period.

### 2.2. Mean Size of Particles

In this study, the SC particles had smaller average diameters when compared to the SDC particles at the beginning and after 90 days of storage (Table 3). This was due to the coating of the spray-dried particles with fat, which resulted in a larger average diameter. The SC and SDC samples had average sizes that changed over 90 days; this increase in particle size may have been due to a change in the morphology or aggregation of the particles during storage. Studies have shown that certain kinds of solid lipid microparticles undergo an appreciable shape change after their formation, as a result of alterations in their polymorphic form, e.g., when the lipid phase undergoes a transition from the α-form to the β-form [33,34]. In addition, this increase in particle size over time can be explained by the use of a heterogeneous fat as carrier, which promotes the aggregation process due to some triacylglycerols that are eventually in liquid form. Process parameters—especially atomisation pressure and temperature—influence particle size [11,35,36]. In addition to the process parameters, the type of active material, the lipid carrier, and the design of the atomiser can also cause considerable size variations [37]. Pelissari et al. [26] found results similar to those in this work when lycopene was immobilised in vegetable fat using SC technology. On the other hand, Fadini et al. [13] found lower values (24.1–25.8 µm) than in the present study when covering particles of fish oil and sacha inchi produced by SD.

According to Pedroso et al. [38,39], particle size is an important parameter to consider for food applications, due to large particles conferring a sandy sensation in the mouth that can lead to consumer rejection of the product. For this reason, these authors suggested that particle size should be smaller than 100 μm in order to avoid negative effects on texture and, thus, on the sensory acceptance of products to which the particles are added. In this context, particles produced under the same conditions applied in this study should be applied to solid or crispy foods, such as cereal bars. Another option is to change the operation conditions and the *RH* of storage, aiming to reduce particle size.

Despite the fact that the particles produced with the smallest concentrations of carotenoids (i.e., SC-20 and SDC-5) displayed slightly better performance in terms of the stability of these compounds, the ones with the highest concentrations of carotenoids (i.e., SC-40 and SDC-15) were chosen for the subsequent assays (i.e., morphology, oxidative stability by the Rancimat method, and release of carotenoids during simulated digestion).

### 2.3. Morphology

**Figure 1:**
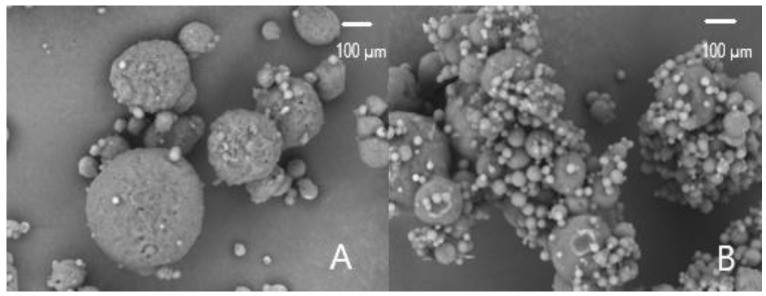
Scanning electron micrographs (1000× magnification) of SC-40 (**A**) and SDC-15 (**B**). SC-40: particles produced in the spray-chiller with 40% extract; SDC-15: particles produced in the spray-chiller with 15% spray-dried particles.

Photomicrographs of SC-40 and SDC-15 particles were obtained via scanning electron microscopy (SEM; 1000× magnification) and are represented in Figure 1A,B, respectively. The SC-40 treatment presented particles that were spherical in shape with agglomerations, variable diameters, and a rough surface with some pores, but without cracks. It is important to produce spherical particles to favour the flow properties and facilitate powder application. Likewise, the agglomeration of particles can also positively contribute to the application of the powder by reducing dust formation. However, particles with rough surfaces can have a reduced flow and indicate the occurrence of a heterogeneous lipid matrix [26]. In addition, particles with pores can expose carotenoids to oxygen, which is a pro-oxidant factor, thereby reducing the functionality of the particles.

The SDC-15 treatment showed a smooth surface with a high level of agglomeration. This may have been related to the complex carrier matrix formed by the combination of hydrogenated vegetable fat and gum Arabic, as well as its water-holding capacity. Incomplete solidification of vegetable fat can occur and lead to the formation of partially melted microparticles; in addition, the collision of microparticles with a high moisture content can also occur, favouring agglomeration. On the other hand, Fadini et al. [13], when covering fish oil and sacha inchi oil particles, observed spherical and polydisperse structures without agglomeration, which could be attributed to the high melting point of the lipid wall. The agglomeration of particles can lead to the formation of structures that no longer have a spherical shape. These external structures can protect the internal ones from environmental conditions, providing additional protection to the encapsulated compounds. Indeed, microparticle morphology can be influenced by operational parameters, which include the temperature, solvents used, feed material composition, and drying rate [40,41].

### 2.4. Oxidative Stability Index by the Rancimat Method

The Rancimat method is widely used to investigate the oxidative stability of oil/lipid-containing samples [42]. Considering that the free extract had oil, and that all particles had oil or fat in their formulations, we studied the oxidative stability of these materials using the Rancimat technique; the results are shown in Table 4. For this evaluation, the following samples were analysed: free extract (as control), SC (spray-chilled particles with 40% raw extract), and SDC (coated particles with 15% spray-dried particles).

According to the results displayed in Table 4, the encapsulation by SC and the coating of the spray-dried particles by SC increased the extract’s oxidative stability in 0.11 and 32.88 h, respectively. These results allow us to infer that the particles have greater oxidative stability than the pure extract, since the pure extract contained sunflower oil, which is rich in unsaturated fatty acids that are very susceptible to oxidation. Sunflower oil is exposed in the free extract, while in the particles it appears to be protected by the carrier. Similar results were found by Santos et al. [43] when they analysed the oxidative stability of tucumã oil (free and encapsulated), which is also a source of unsaturated fatty acids and carotenoids.

In the particles produced in the chiller, sunflower oil was diluted with vegetable fat, which has a high melting point and is rich in saturated fatty acids that are less susceptible to oxidation, but this result was inferior to that for the coated particles because the Rancimat test was performed at a high temperature (120 °C) and the exposure to high-flow oxygen caused an increased surface area and morphological changes, as well as promoting exposure of the lipid matrix and carotenoids, which led to rapid degradation of the carotenoids. Moreover, in those particles produced in the dryer and covered in the chiller, the oil content was lower and was covered by gum Arabic and by the high melting point of the fat, ensuring its stability. In addition, thickening of the wall material and the absence of pores reduced the area of oxygen diffusion and made it possible to form stable systems and effectively protect the encapsulated material. This result is consistent with that obtained for total carotenoid retention (Section 2.1). Pinho et al. [15] found similar results to those of the present study when encapsulating the carotenoid-rich ethanolic extract from guaraná peel using SC and SDC techniques.

### 2.5. Release of Carotenoids from the Particles during Simulated Digestion

The presence of nutrients in foods is commonly assessed by determining their concentrations in products. However, foods go through a series of processes before reaching systemic circulation, which can affect the concentrations and even the chemical forms of food components. One of these processes is gastrointestinal digestion, which involves a series of enzymatic steps that can cause changes in some elements and compounds in the diet and favour hydrolysis and/or the formation of complexes [44].

The initial concentration of carotenoids ($c_{0}$, µg/g) in SC-40 microparticles was 237.9 µg/g, while SDC-15 contained 28.5 µg/g of carotenoids. The SC-40 microparticles were produced in the spray-chiller with 40% extract and with higher values of $c_{0}$. SDC-15 was produced via combined SD and SC methods, resulting in a higher solid barrier, but with lower values of $c_{0}$.

**Figure 2:**
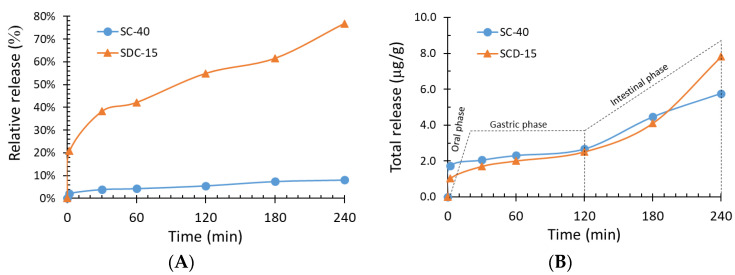
Relative (**A**) and total (**B**) release of carotenoids during simulated digestion.

The relative release (RR, %) and total release (TR, µg/g) of carotenoids varied for each case study, as shown in Figure 2A,B, respectively.

The RR index of SDC-15 microparticles was higher than that of SC-40 microparticles. The addition of spray-dried particles in the SDC method probably led to the release of some of the carotenoids in the gastric phase as a result of the enzymatic degradation of gum Arabic, possibly caused by the presence of SD particles on the surface. In contrast, the lower RR of carotenoids in SC-40 microparticles indicates a more compact microcapsule, with a more efficient barrier to the diffusion of carotenoids.

Despite the $c_{0}$ in SC-40 microparticles being 10 times higher than that in SDC-15 microparticles, the TR was similar in all digestion steps. These profiles provide extra evidence that SC microparticles have a more compact barrier, inhibiting the release of carotenoids in digestive fluids.

The RR of carotenoids from the particles after simulated oral, gastric, and intestinal digestion can be seen in Figure 2A,B. After the oral phase, 0.7% and 4.3% of the carotenoids in the SC and SDC particles, respectively, were released. The release of carotenoids in saliva from SC particles was not expected, because these structures are composed exclusively of lipid compounds that do not dissolve in saliva (aqueous material); in addition, there is no digestion of lipid compounds in this phase. Thus, the small release must have been due to diffusion or to the carotenoids present on the surface of the particles, i.e., the matrix type. Likewise, the release of carotenoids from the SDC particles was also not expected; however, as gum Arabic has a higher solubility of 50% (*w*/*v*) in water at 25 °C [45,46], some of it may have solubilised and released some of the carotenoids. Furthermore, the release of some of the carotenoids present in SDC particles in the saliva suggests that they may have remained on the surface of the microparticles, which we sought to avoid with the coating of spray-dried particles.

A considerable increase in carotenoids was observed after gastric digestion, mainly for the SDC particles, indicating their gradual release from the particles in the oral and gastric phases. In addition, this result indicates that a considerable amount of pigment had withstood the stomach’s acidic conditions, which is interesting since most carotenoids are very sensitive to low pH values.

The reduced levels of carotenoids released at the end of the intestinal phase suggest that one or both of the following possibilities occurred: In the SDC particles, some of the carotenoids may have remained emulsified with gum Arabic and bile salts after centrifugation, as only the supernatant was analysed. The second possible mechanism is that, for both treatments, pigments may have been partially degraded during the gastric and intestinal stages, as the carotenoids undergo rapid degradation when exposed to some enzymes, oxygen, light, and acidic conditions. According to Christophersen et al. [47], some types of solid lipid particles can release bioactive compounds, regardless of the degradation of lipase by diffusion processes, corroborating the results of the present study.

The RR of carotenoids from the SC particles was low (2.4%) compared to the SDC particles (32.6%). These results can be attributed to the polarity of the carriers used in both particles. Gum Arabic is highly hydrophilic and, according to Whorton [48], processes that employ hydrophilic carriers generally trigger a faster release of the active ingredient from the particles. On the other hand, particles produced with highly hydrophobic compounds—such as fats and waxes—as carriers delay the release of the active compounds/nucleus. In fact, the release of active ingredients from particles obtained through SC can occur via erosion and leaching of the matrix. In addition, depending on the type and concentration, some surfactants can dramatically affect the dissolution of the matrix [12]; however, surfactants were not used in the SC particles. Other factors that may have enabled the low release of carotenoids from the SC particles are the low *EE* with the presence of carotenoids on the surface of the particles, causing their degradation, and/or carotenoids being degraded in the acidic conditions of the stomach.

Santos et al. [43] reported a similar release behaviour of carotenoids from spray-dried particles loaded with carotenoid-rich tucumã (*Astrocaryum vulgare* Mart.) oil in simulated gastrointestinal conditions. At the beginning of the gastric phase, a considerable amount of carotenoids was released from the particles (21%), and the release gradually increased until the end of the gastric stage (34%). After intestinal digestion, the release of carotenoids decreased to 9%, following the same trend observed in this study. Donhowe et al. [49] reported 62.6% release of β-carotene after evaluating the in vitro digestion of spray-dried particles containing carotenoids and maltodextrin (also a hydrophilic carrier).

It is worthwhile to note that several factors can affect the release of bioactive compounds from particles during static ‘in vitro’ digestion assays, such as the molecular weight of the bioactive compound, the thickening and diffusion properties of the wall material, and bioactivity and carrier solubility. As expected, due to the high water solubility of gum Arabic, SDC particles released a higher percentage of carotenoids than SC particles in all digestion phases, despite having the lowest concentrations of these bioactive compounds, evidencing that the SDC particles with a double layer showed greater release of carotenoids in the digestive system in vitro.

## 3. Materials and Methods

### 3.1. Materials

Pumpkin peel from *Cucurbita moschata* Dusch. was used for the production of carotenoid-rich extracts. This peel was donated by the company Doces Frutas de Minas (Caldas, MG, Brazil). Gum Arabic (Nexira, Somerville, MA, USA) was used as the carrier for obtaining particles by SD. Al Home P54 hydrogenated vegetable fat with a melting point of 54 °C (Cargill, Itumbiara, Goiás, Brazil) was used as a carrier to obtain the particles by SC, and this same material was also used to cover part of the particles obtained in the spray-dryer. Petroleum ether and magnesium chloride (MgCl_2_·6H_2_O) were acquired from Synth (Diadema, Brazil). Ethanol (purity 99.5%) was purchased from Êxodo Científica (Sumaré, Brazil).

### 3.2. Production of Carotenoid-Rich Pumpkin Peel Extract

The raw peel was cut into small slices using a domestic chopper. The slices were placed on trays and dried in a convective oven (Marconi, MA035/1152, Piracicaba, Brazil) at 40 °C for 18 h and stored at −20 °C until further analysis. A knife miller (Marconi, MA340, Piracicaba, Brazil) was used to crush the dried peel, producing the pumpkin peel flour. This flour was sieved with an 80-mesh sieve. The carotenoids were extracted from the pumpkin peel as described by Ishida and Chapman [50], with some modifications, using absolute ethanol solution as a solvent at a ratio of 1:10 (peel:solvent, *w*/*w*). The use of ethanol as the solvent was based on the fact that it is recognised as safe and obtained from renewable sources. The extraction was carried out in a shaker at 40 °C for 2 h [51]. After this step, 3% sunflower oil was added to the extract relative to the mass of ethanol present in the ethanolic extract, and the mixture was concentrated to one-third of its initial volume using a rotary evaporator (TE-211, Tecnal, Piracicaba, SP, Brazil) at 48 °C [52]. The concentration of the oil was selected after considering the liquid–liquid equilibrium for a system composed of sunflower oil and ethanol [53]. The process was carried out in the absence of light to minimise the degradation of the carotenoids.

### 3.3. Production of Microparticles by Spray-Drying and Coating by Spray-Chilling

For the production of SDC microparticles, four operations were applied—(i) the production of an O/A emulsion; (ii) emulsion homogenisation at high pressure; (iii) encapsulation of this homogenised emulsion by SD; and (iv) coating of spray-dried particles by SD—as shown in Table 5.

An emulsion of ethanolic extract and gum Arabic solution (20% *w*/*w*) at a 1:2 ratio (mL of concentrated extract:mL of gum Arabic solution) was prepared by stirring in an Ultra-Turrax^®^ IKA T25 (Labotechnic, Staufen, Germany) at 10,000 rpm for 3 min [54]. Soon after this process, this emulsion was homogenised at high pressure in a M-110 Y microfluidiser (Microfluidics, Newton, MA, USA) at 100 MPa for one cycle [55].

The obtained emulsion was atomised in a spray-dryer (Model MSD 1.0, Labmaq do Brasil, Ribeirão Preto, Brazil), according to the conditions described by Rocha, Fávaro-Trindade, and Grosso [56], with slight modifications to the drying temperature. The conditions were as follows: 1.2 mm double-spray nozzle, inlet air temperature of 130 °C with an air-drying speed of 2.5 m/s, feed flow of 10 mL/min guaranteed by a peristaltic pump model Labmaq OS-1 (Labmaq do Brasil, Ribeirão Preto, SP, Brazil), and air pressure of 823.8 kPa. During the drying procedure, the emulsion fed to the dryer was kept under magnetic stirring. The obtained powder was stored in a glass jar covered with aluminium foil and stored in a freezer at −20 °C until use.

The spray-dried microparticles were coated using the same equipment (model MSD 1.0 atomiser, from Labmaq de Brasil, Ribeirão Preto, SP, Brazil), using the operational conditions described by Pelissari et al. [26], with some modifications. Suspensions were made using 5%, 10%, and 15% spray-dried particles relative to the molten fat content at 64 °C (*w*/*w*) (the fat was kept 10 °C above the melting point). These suspensions were then atomised in a chamber maintained at 13 °C by means of a cold air stream with a 1.2 mm spray nozzle, 98.07 kPa of air pressure, and 40 mL/min feed flow (controlled by a peristaltic pump).

### 3.4. Production of Microparticles by Spray-Chilling

As shown in Table 5, the production of particles by spray-chilling (named SC particles), as well as their powders, was performed in a single step. For the production of SC particles, the same equipment used was used as for SD (model MSD 1.0 atomiser, from Labmaq de Brasil, Ribeirão Preto, SP, Brazil), using the operational conditions described by Pelissari et al. [26], with some modifications. Dispersions with different proportions of carotenoid-rich extract and high-melting-point hydrogenated vegetable fat were prepared using an Ultra-Turrax^®^ IKA T25 (Labotechnic, Staufen, Germany) at 10,000 rpm for 3 min at 64 °C. The mixture was atomised using the equipment coupled with a 1.2 mm spray nozzle, 98.07 kPa of air pressure, a feed flow of 40 mL/min, and a temperature of 13 °C.

#### 3.4.1. Encapsulation Efficiency (*EE*, %)

*EE* was determined as a function of the ratio of total carotenoid mass in the powders ($c_{P})$ to the carotenoid mass contained in the emulsion $\left(c_{E}\right)$ before drying, as indicated by Equation (1). Microparticle production was conducted in triplicate, and the total carotenoids were quantified as described in Section 3.4.3.

(1) $$EE\left(\%\right)=(c_{P}/c_{E})\cdot 100$$

#### 3.4.2. Characterisation of the Microparticles and their Storage Stability

The microparticles were placed in vials and kept in desiccators containing saturated magnesium chloride solution to obtain a storage environment of 33% ± 5% relative humidity (*RH*), at 25 ± 5 °C, for 90 days.

Total carotenoid and colour parameter analyses were performed at 0, 15, 30, 45, 60, 75, and 90 days. Particle size, moisture content, and water activity analyses were performed only at 0 and 90 days after encapsulation.

#### 3.4.3. Quantification of Total Carotenoids

Total carotenoids in powders were determined by a spectrophotometric method, as described by Rodriguez-Amaya [57]. Carotenoids were extracted as described by Pelissari et al. [26], with slight modifications to the solvents used. Carotenoids were extracted from 10 mg of powder, to which was added 1 mL of ethanol, 3 mL of petroleum ether, and 1 mL of deionised water. The mixtures were agitated for 1 min and kept in an ultrasound bath (USC-1400, Unique, Indaiatuba, Brazil) for 10 min. They were then centrifuged at 4930× *g* for 5 min, and petroleum-ether-rich phases were transferred to cuvettes and analysed at 450 nm in a spectrophotometer (Genesys 10S Thermo Scientific, São Paulo/SP, Brasil). Petroleum ether solvent was used as a blank sample. The quantification of total carotenoids was determined according to Equation (2):

(2) $$c=\frac{Abs\cdot V\cdot 10^{4}}{Abs_{1cm}^{1\%}\cdot \;m}$$

where *Abs* is the absorbance, *V* is the final volume (mL), $Abs_{1cm}^{1\%}$ is the β-carotene absorption coefficient in petroleum ether (2592 cm^−1^), and *m* is the sample mass (g).

Powders were periodically evaluated for carotenoid concentration and instrumental colour after 0, 15, 30, 45, 60, 75, and 90 days of storage in desiccators kept in a dark room. The quantification of total carotenoids in the powders was performed in triplicate, as described in Section 3.4.3, and the amount of total carotenoids at day 0 (*t* = 0 day) was considered as 100%. The degradation constant k (μg·g^−1^·s^−1^) (Equation (3)) and the half-life $t_{1/2}$ (s) (Equation (5)) were determined according to the zero-order kinetics of degradation, as follows:

(3) $$-\frac{\partial c}{\partial t}=k$$

(4) $$c=c_{0}-kt$$

(5) $$t_{1/2}=\frac{c_{0}}{2k}$$

where $c_{0}$ (µg/g) is the amount of total carotenoids at day 0 (*t* = 0 day) and c (µg/g) is the amount of total carotenoids determined over the timeline.

#### 3.4.4. Carotenoid Retention

Carotenoid retention (CR, %) was determined according to the following equation:

(6) $$CR=\left(c_{90}/c_{0}\right)\cdot 100$$

where $c_{90}$ (μg/g) is the carotenoid concentration after 90 days of storage and $c_{0}$ (μg/g) is the initial carotenoid concentration in the microparticles.

#### 3.4.5. Instrumental Colour Parameters

Instrumental colour parameters of the powders were determined using a MiniScan XE Plus (Hunter Lab, Reston, Virginia, USA) to establish a correlation between the stability and colour changes after total carotenoid degradation. The parameters chroma ($C^{*}$), and hue angle (h°) were defined by Equations (7) and (8). Chroma is considered to be the quantitative attribute of colourfulness, while hue is the qualitative attribute. The total color difference (Δ*E*) on day 90 of storage was determined using Equation (9).

(7) $$C^{*}={\left(a^{2}+b^{2}\right)}^{1/2}$$

(8) h°=arctan(b/a)

(9) $$\Delta E={\left(\Delta L^{2}+\Delta a^{2}+\Delta b^{2}\right)}^{1/2}$$

where $\Delta L=\left(L_{0\;day}^{*}-L_{day\;90}^{*}\right)$, $\Delta a=\left(a_{0\;day}^{*}-a_{day\;90}^{*}\right)$, and $\Delta b=\left(b_{0\;day}^{*}-b_{day\;90}^{*}\right)$ are the L*, a*, and b* values of the powder surface measured according to the CIELAB system, respectively.

#### 3.4.6. Particle Size

The particle size distribution and the volumetric mean diameter were evaluated at day 0 and after 90 days of storage, using a laser diffraction particle analyser (Shimadzu Sald-201V, Kyoto, Japan). Before the analyses, the powders were added to ethanol (Synth, Diadema, SP, Brazil) for better dispersion of the material.

### 3.5. Analysis of Selected Microparticles

To further analyse the characteristics of the microparticles, two formulations were selected. Samples SC-40 and SDC-15 were chosen, mainly based on the results for their total carotenoid contents and their potential to be used as food colourings. They were evaluated in terms of their morphology, oxidative stability, and release of carotenoids during in vitro digestion, as described in the following sections.

#### 3.5.1. Morphology

SC and SDC microparticles were placed on a double-sided carbon adhesive tape (Ted Pella Inc., Redding, CA, USA); they were coated with gold and analyzed using SEM (Benchtop Microscope Hitachi TM 300, Tokyo, Japan). SEM images of the microparticles were captured at an accelerating voltage of 15 Kv.

#### 3.5.2. Oxidative Stability Index by the Rancimat Method

Accelerated oxidation tests were carried out using Rancimat equipment (model 873, Metrohm, Herisau, Switzerland). Samples were subjected to heating under a purified airflow rate of 20 L/h at 120 °C. The induction time of the sample was used as the oxidative stability index (OSI), measured in hours. Four grams of free extract and one gram of powders were used for this assay.

#### 3.5.3. Total Release and Relative Release of Carotenoids during In Vitro Digestion

This study aimed to investigate the effect of the microencapsulation method on the release of carotenoids from particles by performing an in vitro simulated digestion test. The composition of simulated gastrointestinal fluids followed the recommended electrolytic concentration described by Minekus et al. [58]. Approximately 0.5 g of microparticles was added to 1.5 mL of distilled water and 2 mL of simulated salivary fluid (SSF) for 5 min. Subsequently, about 4 mL of simulated gastric fluid (SGF) was added to the tubes, followed by adjustment of the pH to 3 using HCl (5 M) and the addition of 0.2 mL of pepsin solution (2000 U/mL). The tubes were incubated at 37 °C under agitation at 200 rpm for 120 min. After that, simulated intestinal fluid (SIF) was added to the tubes, and the pH was changed to 7 using NaOH (1 M). Approximately 2 mL of pancreatin solution (100 U/mL) and 1 mL of bile salts (10 mM) were added to the mixture; thus, the tubes were incubated under the same conditions described. Samples were evaluated after 30, 60, and 120 min for SGF and 130, 180, and 240 min for SIF.

To quantify the carotenoids in SSF, SGF, and SIF, approximately 0.5 mL of supernatant was first centrifuged (2935× *g*, 5 min). The quantification of the carotenoids released was performed as described in Section 3.4.3.

The relative release RR (µg/µg) and total release TR (µg/g) of the carotenoids were evaluated during the digestion steps.

Analysis of the quantification of carotenoids measured the concentration of carotenoids ($c_{i}$, µg/g) and the mass of the solvent ($m_{f,i}$, g) in the solvent phase. The mass of carotenoids ($m_{c,i}$, µg) in the microparticles was evaluated according to Equation (10), in each time step i:

(10) $$m_{c,i}=c_{i}\cdot m_{f,i}$$

Given the initial concentration of carotenoids in the microparticles ($c_{0}$, µg/g) and the mass of microparticles ($m_{s,i}$, g) at digestion step i, the RR (µg/µg) was determined by Equation (11). The RR index is the cumulative mass of carotenoids released during digestion ($m_{c,i}$, µg), relative to the initial mass of carotenoids ($m_{s,i}\cdot c_{0}$).

(11) $$RR_{i}=\sum_{0}^{i}\frac{m_{c,i}}{m_{s,i}c_{0}}$$

TR (µg/g) is the ratio between the mass of carotenoids released ($m_{c,i}$, µg) and the mass of microparticles ($m_{s,i}$, g) at step i, as shown in Equation (12):

(12) $$TR_{i}=\sum_{0}^{i}\frac{m_{c,i}}{m_{s,i}}$$

### 3.6. Statistical Analysis

All experiments were performed in triplicate. Statistical analyses, including ANOVA and the Tukey–Kramer honestly significant difference test for comparisons of means, were conducted using the statistical program SAS (Statistic Analysis System) version 9.2; *p* < 0.05 was considered to be statistically significant.

## 4. Conclusions

In this study, the encapsulation process was shown to be an effective approach to provide pigment stability with a higher *EE*, ranging from 82.9% to 100%. Double-layer particles showed increased carotenoid retention (60.8–89.7%), prolonged half-life, and stable pigmentation with a smaller colour difference (3.48–6.39) on day 90 of storage. The mean diameter exhibited an increase of 33% for SC particles and 15% for SDC particles after 90 days; however, these particles can be applied to food products. The kinetics of carotenoid degradation followed zero-order kinetics for most samples, possibly caused by oxygen permeation in the microparticles’ core. Micrographs showed SDC samples with a smooth surface and heterogeneous sizes, while micrographs of SC samples showed particles that were spherical in shape with agglomerations, variable diameters, and a rough surface with some pores. SDC particles increased the oxidative stability of carotenoids and released more carotenoids than the chilled particles during digestion; however, they had lower concentrations of these compounds. SC and, in particular, the combination of SD and SC, represent promising alternatives for producing carotenoid-rich powders, representing opportunities to add value to a byproduct of the food industry and making it possible to produce a natural dye with the potential to be used in food, pharmaceuticals, and other materials. Further research should be conducted to evaluate the application of these particles in food products.

## Figures and Tables

**Table 1:** Encapsulation efficiency (*EE*, %), retention index (CR, %) after 90 days of powder storage, initial concentration ($c_{0}$, µg/g), and regression coefficients (*k*, *t*_1/2_, and *R*^2^) of zero-order reaction fitting.

Trial	*EE* (%)	CR (%)	$c_{0}$ (µg/g)	k (μg·g^−1^·s^−1^)	$t_{1/2}$ (day)	$R^{2}$
SC-20	82.9 ± 8.4 ^B^	65.9 ± 4.4 ^A^	66.4	0.205	161.4	0.866
SC-30	97.4 ± 4.0 ^A^	51.1 ± 8.4 ^C^	133.97	0.840	79.7	0.990
SC-40	94.3 ± 6.7 ^A^	59.9 ± 3.2 ^B^	237.96	1.096	108.6	0.977
SDC-5	96.8 ± 13.7 ^A^	89.7 ± 7.0 ^A^	8.74	0.017	256	0.857
SDC-10	100.8 ± 4.5 ^A^	66.9 ± 6.6 ^B^	16.8	0.054	154.3	0.825
SDC-15	95.2 ± 15.4 ^A^	60.8 ± 7.2 ^C^	28.5	0.094	150.8	0.946

Results are means ± SD (*n* = 3), values with different capital letters differ significantly (*p* < 0.05). SC: microparticles obtained by spray chilling; SDC: microparticles obtained by spray drying and chilling combination. The numerical suffix denotes the proportion of core/wall material of each formulation.

**Table 2:** Values of instrumental colour parameters during storage of the microparticles measured on days 0 and 90 of storage, at 25 °C and 33% *RH*.

Trial	Luminosity $\left(L^{*}\right)$ Day 0	Luminosity $\left(L^{*}\right)$ Day 90	Chroma $\left(C^{*}\right)$ Day 0	Chroma $\left(C^{*}\right)$ Day 90	Hue Angle (h°) Day 0	Hue Angle (h°) Day 90	Color Difference (ΔE)
SC-20	77.4 ± 3.3 ^a^	76.5 ± 1.5 ^b^	35.2 ± 0.18 ^c^	33.9 ± 0.99 ^c^	57.07 ± 0.13 ^e^	56.77 ± 0.01 ^e^	4.21 ± 0.2 ^B^
SC-30	78.9 ± 0.8 ^a^	74.1 ± 1.4 ^b^	42.06 ± 3.52 ^c^	40.91 ± 2.77 ^c^	55.97 ± 0.07 ^e^	56.34 ± 0.15 ^e^	7.06 ± 1.3 ^A^
SC-40	78.7 ± 0.08 ^a^	74.2 ± 1.8 ^b^	53.4 ± 1.11 ^c^	47.5 ± 0.28 ^d^	55.72 ± 0.11 ^e^	55.96 ± 0.22 ^e^	7.45 ± 2.2 ^A^
SDC-5	84.5 ± 1.6 ^a^	78.2 ± 1.5 ^b^	22.45 ± 0.05 ^c^	22.2 ± 0.89 ^c^	57.36 ± 0.12 ^f^	86.68 ± 0.0 ^e^	6.39 ± 0.1 ^A^
SDC-10	79.7 ± 1.12 ^a^	76.7 ± 1.5 ^b^	28.95 ± 1.09 ^c^	29.16 ± 0.74 ^c^	57.0 ± 0.02 ^e^	57.16 ± 0.03 ^e^	3.53 ± 0.4 ^B^
SDC-15	79.4 ± 0.02 ^a^	77.4 ± 0.4 ^b^	35.51 ± 0.18 ^c^	32.7 ± 0.22 ^d^	56.64 ± 0.02 ^e^	56.94 ± 0.03 ^e^	3.48 ± 0.2 ^B^

Results are means ± SD (*n* = 3). Different capital letters in a column represent a significant difference (*p* < 0.05) between formulations. The different lowercase letters (a–f) in a row indicate a significant difference among formulations by the Tukey test at the 5%. SC: microparticles obtained by spray chilling; SDC: microparticles obtained by spray drying and chilling combination. The numerical suffix denotes the proportion of core/wall material of each formulation.

**Table 3:** Volume-weighted mean diameter (*D_4,3_*) of the microparticles measured on days 0 and 90 of storage, at 25 °C and 33% *RH*.

Trial	$D_{0\_day}$ (µm)	$D_{day\_90}$ (µm)
SC-20	70.9 ± 7.0 ^b^	99.5 ± 6.2 ^a^
SC-30	72.1 ± 6.0 ^b^	109.4 ± 10.8 ^a^
SC-40	72.1 ± 5.0 ^b^	112.7 ± 17.3 ^a^
SDC-5	110.7 ± 7.8 ^b^	117.5 ± 9.7 ^a^
SDC-10	106.9 ± 6.3 ^b^	122.9 ± 6.3 ^a^
SDC-15	97.7 ± 2.2 ^b^	132.2 ± 13.9 ^a^

The different lowercase letters in a row indicate a significant difference among formulations by the Tukey test at the 5%. SC: microparticles obtained by spray chilling; SDC: microparticles obtained by spray drying and chilling combination. The numerical suffix denotes the proportion of core/wall material of each formulation.

**Table 4:** Oxidation induction times of the non-encapsulated extract and powders obtained by spray-chilling (SC) and -coating (SDC), as evaluated by Rancimat at 120 °C.

Trial	Induction Time (h)
Non-encapsulated extract	0.03 ± 0.01 ^C^
SC-40	0.13 ± 0.02 ^B^
SDC-15	32.91 ± 0.0 ^A^

Values with the same capital letter are not statistically different (*p* > 0.05). SC: microparticles obtained by spray chilling; SDC: microparticles obtained by spray drying and chilling combination. The numerical suffix denotes the proportion of core/wall material of each formulation.

**Table 5:** Description of the components used in the production of powders/particles.

Formulation	Core (%)	Carrier Material (%)
	Free extract	Vegetable fat
SC-20	20	80
SC-30	30	70
SC-40	40	60
	SD microparticles	Vegetable fat
SDC-5	5	95
SDC-10	10	90
SDC-15	15	85

SD: spray-dried particles/powders produced with a 1:2 extract:gum Arabic solution (20 g/100 g) ratio; SC: spray-chilled particles/powders; SDC: spray-dried particles coated by spray-chilling.

## Data Availability

The data presented in this study are available upon request from the corresponding author.

## Sample Availability

Samples of the compounds are no available from the authors.

## References

[1] Siegmund B., Murkovic M. (2004). Changes in chemical composition of pumpkin seeds during the roasting process for production of pumpkin seed oil (Part 2: Volatile compounds). Food Chem..

[2] Norfezah M.N., Hardacre A., Brennan C.S. (2011). Comparison of waste pumpkin material and its potential use in extruded snack foods. Food Sci. Technol. Int..

[3] Fu L., Wang X. (2012). Extraction of carotenoids from pumpkin peel. Chin. Agric. Sci. Bull..

[4] Nuerbiya Y., Ayinuer R., Abdulla A. (2014). Optimization of extraction pigment from pumpkin skin product’s stability. Food and Ferment. Ind..

[5] Milani A., Basirnejad M., Shahbazi S., Bolhassani A. (2017). Carotenoids: Biochemistry, pharmacology and treatment. Br. J. Pharmacol..

[6] Xianquan S., Shi J., Kakuda Y., Yueming J. (2005). Stability of lycopene during food processing and storage. J. Med. Food.

[7] Chranioti C., Nikoloudaki A., Tzia C. (2015). Saffron and beetroot extracts encapsulated in maltodextrin, gum Arabic, modified starch and chitosan: Incorporation in a chewing gum system. Carbohydr. Polym..

[8] Domian E., Brynda-kopytowska A., Oleksza K. (2015). Rheological properties and physical stability of o/w emulsions stabilized by OSA starch with trehalose. Food Hydrocoll..

[9] Janiszewska E. (2014). Microencapsulated beetroot juice as a potential source of betalain. Powder Technol..

[10] Kolanowski W., Ziolkowski M., Weissbrodt J., Kunz B., Laufenberg G. (2006). Microencapsulation of fish oil by spray drying—Impact on oxidative stability. Part 1. Eur. Food Res. Technol..

[11] Favaro-trindade C.S., Okuro P.K., Matos F.E., Mishra M. (2015). Encapsulation via Spray Chilling/Cooling/Congealing. Handbook of Encapsulation and Controlled Release.

[12] Okuro P.K., Eustáquio de Matos F., Favaro-Trindade C.S. (2013). Technological challenges for spray chilling encapsulation of functional food ingredients. Food Technol. Biotechnol..

[13] Fadini A.L., Alvim I.D., Ribeiro I.P., Ruzene L.G., da Silva L.B., Queiroz M.B., de Oliveira Miguel A.M.R., Chaves F.C.M., Rodrigues R.A.F. (2018). Innovative strategy based on combined microencapsulation technologies for food application and the influence of wall material composition. LWT-Food Sci. Technol..

[14] Arslan-Tontul S., Erbas M. (2017). Single and double layered microencapsulation of probiotics by spray drying and spray chilling. LWT-Food Sci. Technol..

[15] Pinho L.S., de Lima P.M., de Sá S.H.G., Chen D., Campanella O.H., da Costa Rodrigues C.E., Favaro-Trindade C.S. (2022). Encapsulation of Rich-Carotenoids Extract from Guaraná (*Paullinia cupana*) Byproduct by a Combination of Spray Drying and Spray Chilling. Foods.

[16] Mascarenhas J.M.O. (1998). Dyes in Foods: Perspectives, Uses and Restrictions. Master’s Thesis.

[17] Mota I. (2016). Artificial Dyes: Health Risks and Need for Revision of Brazilian Regulations. Undergraduate Thesis.

[18] Schiozer A.L., Barata L.E.S. (2007). Stability of dye and pigments of vegetable origin—A review. Rev. Fitos.

[19] Rodriguez-Amaya D.B. (2002). Effects of processing and storage on food carotenoids. Sight Life Newslett..

[20] Cutrim C.S., Alvim I.D., Cortez M.A.S. (2019). Microencapsulation of green tea polyphenols by ionic gelation and spray chilling methods. J. Food Sci. Technol..

[21] Gamboa O.D., Gonçalves L.G., Grosso C.F. (2011). Microencapsulation of tocopherols in lipid matrix by spray chilling method. Procedia Food Sci..

[22] Sartori T., Consoli L., Hubinger M.D., Menegalli F.C. (2015). Ascorbic acid microencapsulation by spray chilling: Production and characterization. LWT-Food Sci. Technol..

[23] Lima P.M., Rubio F.T.V., Silva M.P., Pinho L.S., Kasemodel M.G.C., Favaro-Trindade C.S., Dacanal G.C. (2019). Nutritional Value and Modelling of Carotenoids Extraction from Pumpkin (Cucurbita Moschata) Peel Flour By-Product. Int. J. Food Eng..

[24] Provesi J.G., Dias C.O., Amante E.R. (2011). Changes in carotenoids during processing and storage of pumpkin puree. Food Chem..

[25] Rodriguez-Amaya D.B. (1999). Changes in carotenoids during processing and storage of foods. Arch. Latino Am. Nutr..

[26] Pelissari J.R. (2016). Production of solid lipid microparticles loaded with lycopene by spray chilling: Structural characteristics of particles and lycopene stability. Food Bioprod. Process..

[27] Santos P.P., Paese K., Guterres S.S., Pohlmann A.R., Costa T.H., Jablonski A., Flôres S.R., Rios A.A. (2015). Development of lycopene-loaded lipid-core nanocapsules: Physicochemical characterization and stability study. J. Nanoparticle Res..

[28] Pathare P.B., Opara U.L., Al-Said F.A.J. (2013). Colour measurement and analysis in fresh and processed foods: A review. Food Bioprocess Technol..

[29] Oliveira S.M., Ramos I.N., Brandão T.R.S., Silva C.L.M. (2015). Effect of air-drying temperature on the quality and bioactive characteristics of dried galega kale (*Brassica oleracea* L. Var. Acephala). J. Food Process. Preserv..

[30] Mcclements D.J. (2002). Theoretical prediction of emulsion color. Adv. Colloid Interface Sci..

[31] Rentfrow G., Linville M.L., Stahl C.A., Olson K.C., Berg E.P. (2004). The effects of the antioxidant lipoic acid on beef longissimus bloom time. J. Anim. Sci..

[32] Haas K., Obernberger J., Zehetner E., Kiesslich A., Volkert M., Jaeger H. (2019). Impact of powder particle structure on the oxidation stability and color of encapsulated crystalline and emulsified carotenoids in carrot concentrate powders. J. Food Eng..

[33] Trotta M., Pattarino F., Ignoni T. (2002). Stability of drug-carrier emulsions containing phosphatidylcholine mixtures. Eur. J. Pharm. Biopharm..

[34] Westesen K., Siekmann B. (1997). Investigation of the gel formation of phospholipid-stabilized solid lipid nanoparticles. Int. J. Pharm..

[35] Di Sabatino M., Albertini B., Kett V.L., Passerini N. (2012). Spray congealed lipid microparticles with high protein loading: Preparation and solid state characterisation. Eur. J. Pharm. Sci..

[36] Maschke A., Becker C., Eyrich D., Kiermaier J., Blunk T., Göpferich A. (2007). Development of a spray congealing process for the preparation of insulin-loaded lipid microparticles and characterization thereof. Eur. J. Pharm. Biopharm..

[37] Matos F.E., Comunian T.A., Thomazini M., Favaro-Trindade C.S. (2007). Effect of feed preparation on the properties and stability of ascorbic acid microparticles produced by spray chilling. LWT-Food Sci. Technol..

[38] Pedroso D.L., Thomazini M., Heinemann R.J.B., Favaro-Trindade C.S. (2012). Protection of Bifidobacterium lactis and Lactobacillus acidophilus by microencapsulation using spray-chilling. Int. Dairy J..

[39] Pedroso D.L., Dogenski M., Thomazini M., Heinemann R.J.B., Favaro-Trindade C.S. (2013). Microencapsulation of Bifidobacterium animalis subsp. lactis and Lactobacillus acidophilus in cocoa butter using spray chilling technology. Braz. J. Microbiol..

[40] Alves A.I. (2017). Morphological characterization of pequi extract microencapsulated through spray drying. Int. J. Food Prop..

[41] Lourenço S.C., Moldão-Martins M., Alves V.D. (2020). Microencapsulation of pineapple peel extract by spray drying using maltodextrin, inulin, and arabic gum as wall matrices. Foods.

[42] Choe E., Min D.B. (2006). Mechanisms and factors for edible oil oxidation. Compr. Rev. Food Sci. Food Saf..

[43] Santos P.D.F., Rubio F.T.V., Balieiro J.C.C.B., Thomazini M., Favaro-Trindade C.S. (2021). Application of spray drying for production of microparticles containing the carotenoid-rich tucumã oil (Astrocaryum vulgare Mart.). LWT-Food Sci. Technol..

[44] Montero P., Calvo M.M., Gómez-Guillén M.C., Gómez-Estaca J. (2016). Microcapsules containing astaxanthin from shrimp waste as potential food coloring and functional ingredient: Characterization, stability, and bioaccessibility. LWT-Food Sci. Technol..

[45] Montenegro M.A., Boiero M.L., Valle L., Borsarelli C.D. (2012). Gum Arabic: More Than an Edible Emulsifier. Products and Applications of Biopolymers.

[46] Yang Y.Y., Chung T.S., Ng N.P. (2001). Morphology, Drug Distribution, and in Vitro Release Profiles of Biodegradable Polymeric Microspheres Containing Protein Fabricated by Double-Emulsion Solvent Extraction/Evaporation Method. Biomaterials.

[47] Christophersen P.C., Birch D., Saarinen J., Isomäki A., Nielsen H.M., Yang M., Strachan C.J., Mu H. (2015). Investigation of protein distribution in solid lipid particles and its impact on protein release using coherent anti-Stokes Raman scattering microscopy. J. Control. Release.

[48] Whorton C. (1995). Factors Influencing Volatile Release from Encapsulation Matrices.

[49] Donhowe E.G., Flores F.P., Kerr W.L., Wicker L., Kong F. (2014). Characterization and in vitro bioavailability of β-carotene: Effects of microencapsulation method and food matrix. LWT-Food Sci. Technol..

[50] Ishida B.K., Chapman M.H. (2009). Carotenoid extraction from plants using a novel, environmentally friendly solvent. J. Agric. Food Chem..

[51] Das S., Bera D. (2013). Mathematical model study on solvent extraction of carotene from carrot. Int. J. Res. Eng. Technol..

[52] D’evoli L., Lombardi-boccia G., Lucarini M. (2013). Influence of Heat Treatments on Carotenoid Content of Cherry Tomatoes. Foods.

[53] Cuevas M.S., Rodrigues C.E.C., Gomes G.B., Meirelles A.J.A. (2010). Vegetable oils deacidification by solvent extraction: Liquid− Liquid equilibrium data for systems containing sunflower seed oil at 298.2 K. J. Chem. Eng. Data.

[54] Xu D., Wang X., Yuan F., Hou Z., Gao Y. (2013). Stability of β-Carotene in Oil-in-Water Emulsions Prepared by Mixed Layer and Bilayer of Whey Protein Isolate and Beet Pectin. J. Dispers. Sci. Technol..

[55] Mutsokoti L., Panozzo A., Pallares Pallares A., Jaiswal S., Van Loey A., Grauwet T., Hendrickx M. (2017). Carotenoid bioaccessibility and the relation to lipid digestion: A kinetic study. Food Chem..

[56] Rocha G.A., Fávaro-Trindade C.S., Grosso C.R.F. (2012). Microencapsulation of lycopene by spray drying: Characterization, stability and application of microcapsules. Food Bioprod. Process..

[57] Rodriguez-Amaya D.B. (2001). A Guide to Carotenoids Analysis in Food.

[58] Minekus M., Alminger M., Alvito P., Ballance S., Bohn T., Bourlieu C., Carrière F., Boutrou R., Corredig M., Dupont D. (2014). A standardised static in vitro digestion method suitable for food-an international consensus. Food Funct..

